# Open Labeled, Randomized, Switch-Over Study of Two Fixed Doses (10/15mg) Of Aripiprazole : To Evaluate its Safety And Efficacy in the Treatment of Indian Patients of Schizophrenia.

**Published:** 2004

**Authors:** A Sarin, J Nagpal, N K Bohra, R C Jiloha, G P Rao, S K Sharma, M Vaishnav, L Vaya, R S Karan, N K Patel, R Patel

**Affiliations:** 1 Consultant Psychiatrist, Vidyasagar Institute of Mental Health and Neurosciences, 1, Institutional Area, Nehrunagar, New Delhi - 110 017; 2 Consultant Psychiatrist, Vidyasagar Institute of Mental Health and Neurosciences, 1, Institutional Area, Nehrunagar, New Delhi - 110 017; 3 Consultant Psychiatrist, B-65 Friends Colony (West), New Delhi - 110 065; 4 Professor & Head, Department of Psychiatry, G. B. Pant Hospital, New Delhi - 110 002; 5 Consultant Psychiatrist, Flat No. 102, Sai Vishnu Apartment, Damalguda, 1-2-26, HYDERABAD - 500 029; 6 Associate Professor, Government Medical College, UDAIPUR - 313 001; 7 Consultant Psychiatrist, 103-104, First Floor, Samvedna Hospital, Below Karnavati Hospital, Ellisbridge, AHMEDABAD - 380 006; 8 Department of Clinical Research, Torrent Research Centre, Village Bhat, Gandhinagar.; 9 Department of Clinical Research, Torrent Research Centre, Village Bhat, Gandhinagar.; 10 Department of Clinical Research, Torrent Research Centre, Village Bhat, Gandhinagar.; 11 Department of Clinical Research, Torrent Research Centre, Village Bhat, Gandhinagar.

**Keywords:** Aripiprazole, Schizophrenia, Switch Study

## Abstract

Aripiprazole is a new anti psychotic with a unique receptor binding profile that combines partial agonistic activity at D2 receptor and 5-HT 1A receptor and potent antagonism at 5-HT 2A receptor. This receptor profile makes it possible for it to act as a dopamine system stabilizer. Based on various short term and long term studies, aripiprazole has been found to be effective in schizophrenia and has no significant adverse effect on QTc prolongation, prolactin, serum lipids, and has a low potential for weight gain. Present study aims to evaluate the efficacy and tolerability of aripiprazole (10-15mg/day) in the treatment of Indian patients of schizophrenia and to see its effect on QTc interval, prolactin levels, serum lipids, plasma sugar and weight gain in these patients. Outpatients with an ongoing/newly diagnosed ICD-10 Schizophrenia (n=136) were randomly assigned to 10 or 15 mg dose of Aripiprazole for a period of six weeks. Clinical response was evaluated by the Positive And Negative Symptoms Scale (PANSS), Clinical Global Impression (CGI) scale and safety was evaluated by observing spontaneously reported adverse events and changes in various laboratory parameters. Switching schizophrenic patients to aripiprazole (10/15 mg) from both conventional and atypical anti-psychotics was safe and well tolerated. Six weeks after switching to aripiprazole, patients showed improvements in PANSS scores (P< 0.001), EPS, prolactin levels and weight over the baseline levels. No difference was seen in the 10 or 15mg dose groups. One hospitalization was reported (due to hepatitis E). Common side effects reported were insomnia, somnolence, nausea, vomiting and diarrhea. Aripiprazole is a safe and effective anti psychotic in Indian patients - both in newly diagnosed, as well as, in patients not responding to or intolerant to other available typical and atypical antipsychotics.


 Indian Journal of Psychiatry, 2004, 46(I)64-71

### Open Labeled, Randomized, Switch-over Study of Two Fixed Doses (10/15mg) of Aripiprazole: To Evaluate its Safety and Efficacy in the Treatment of Indian Patients of Schizophrenia.

**Sarin A**[1], **Nagpal J** [2], **Bohra NK**[3],. **Jiloha R.C**[4], **Rao GP**[5], **Sharma SK**[6], **Vaishnav M** [7].
**Vaya L***[8], **Karan RS**[9], **Patel NK**[10] and **Patel R**[11].

### ABSTRACT:

Aripiprazole is a new anti psychotic with a unique receptor binding profile that combines partial agonistic activity at D2 receptor and 5-HT 1A receptor and potent antagonism at 5-HT 2A receptor. This receptor profile makes it possible for it to act as a dopamine system stabilizer. Based on various short term and long term studies, aripiprazole has been found to be effective in schizophrenia and has no significant adverse effect on QTc prolongation, prolactin, serum lipids, and has a low potential for weight gain. Present study aims to evaluate the efficacy and tolerability of aripiprazole (10-15mg/day) in the treatment of Indian patients of schizophrenia and to see its effect on QTc interval, prolactin levels, serum lipids, plasma sugar and weight gain in these patients. Outpatients with an ongoing/newly diagnosed ICD-10 Schizophrenia (n=136) were randomly assigned to 10 or 15 mg dose of Aripiprazole for a period of six weeks. Clinical response was evaluated by the Positive And Negative Symptoms Scale (PANSS), Clinical Global Impression (CGI) scale and safety was evaluated by observing spontaneously reported adverse events and changes in various laboratory parameters. Switching schizophrenic patients to aripiprazole (10/15 mg) from both conventional and atypical anti-psychotics was safe and well tolerated. Six weeks after switching to aripiprazole, patients showed improvements in PANSS scores ($P<0.001$), EPS, prolactin levels and weight over the baseline levels. No difference was seen in the 10 or 15mg dose groups. One hospitalization was reported (due to hepatitis E). Common side effects reported were insomnia, somnolence, nausea, vomiting and diarrhea. Aripiprazole is a safe and effective anti psychotic in Indian patients — both in newly diagnosed, as well as, in patients not responding to or intolerant to other available typical and atypical antipsychotics.

*Key words:* Aripiprazole, Schizophrenia, Switch Study.

### INTRODUCTION

A new concept for the action of antipsychotics drugs that do not induce motor side effects is dopamine system stabilization (Stahl 2001; Stahl 2001). The brain normally stabilizes dopamine neurotransmission by attaining a balance between presynaptic and postsynaptic D2 receptor stimulation. These 2 mechanisms act together: presynaptic dopamine receptors are stimulated, and dopamine release at specific post-synaptic sites is turned off, thus reducing excessive dopamine activation in parts of the brain where the concentration is too high yet permitting normal dopamine activity in other parts of the brain. The presynaptic, D2 receptors responsible for regulating dopamine release are less sensitive in detecting dopamine than are postsynaptic D2 receptors, so physiologic neurotransmission continues until dopamine levels build up sufficiently to stimulate the presynaptic D2 receptors, thereby turning off further dopamine release.

In the brain, regional differences may occur in the activity of dopamine neurons and the sensitivity of various presynaptic and postsynaptic D2 receptors to dopamine, especially during disease states and as a consequence of

[1] Consultant Psychiatrist, Vidyasagar Institute of Mental Health and Neurosciences, 1, Institutional Area, Nehrunagar, New Delhi – 110 017,

[2] Consultant Psychiatrist, Vidyasagar Institute of Mental Health and Neurosciences, 1, Institutional Area, Nehrunagar, New Delhi –110 017,

[3] Consultant Psychiatrist, B-65 Friends Colony (West), New Delhi – 110 065,

[4] Professor & Head, Department of Psychiatry, G. B. Pant Hospital, New Delhi – 110 002

[5] Consultant Psychiatrist, Flat No. 102, Sai Vishnu Apartment, Damalguda, 1-2-26, HYDERABAD – 500 029,

[6] Associate Professor, Government Medical College, UDAIPUR – 313 001,

[7] Consultant Psychiatrist, 103-104, First Floor, Samvedna Hospital, Below Karnavati Hospital, Ellisbridge, AHMEDABAD – 380 006, 8-11) Department of Clinical Research, Torrent Research Centre, Village Bhat, Gandhinagar.

* Correspondence: Dr. Lalit Vaya, Department of Clinical Research, Torrent Research Centre, Village: Bhat, Dist: Gandhinagar – 382 428.
Tel. No. 079-3969100, Fax No.:079-3969135 E-mail:vayalalit@torrentpharma.com, rajeshsinghkaran@torrentpharma.com various drug treatments. The concept of dopamine system stabilization is based on preserving or enhancing dopaminergic neurotransmission where it is low ("too cold") and reducing dopaminergic neurotransmission where it is too high ("too hot"). In terms of treating psychosis, the goal is to reduce hyperactive dopamine neurons that mediate psychosis and at the same time enhance underactive dopamine neurons that mediate negative and cognitive symptoms (mesocortical pathway) while preserving physiologic function in dopamine neurons that regulate motor movement and prolactin- all in the same brain at the same time.

When dopamine is dysregulated in psychoses (e.g., schizophrenia), dopamine system stabilizers (DSSs), which bind to D2 receptors in a manner that can be either stimulating or antagonizing, are helpful. These stabilizers are far different from receptor antagonists, which always block the action of dopamine completely and reduce output from D2 receptors ("too cold"), and very different from dopamine itself, which is a full agonist at D2 receptors that creates maximum action (if the amount is high enough), making the receptors "too hot". DSSs, on the other hand, theoretically create the desired dopamine balance. Thus, when dopamine activity is too cold, a DSS increases dopamine output, but to a level not as hot as real dopamine. In the presence of maximum dopamine DSSs reduce the amount until it is "just right".

Pharmacologically, this mechanism is known as partial agonist action, which means activation at low dopaminergic tone and inhibition at high dopaminergic tone, thus stabilizing dopamine output from either direction. Molecularly, the exact mechanism of partial agonist binding to D2 receptors remains somewhat obscure but is hypothesized to exploit differences in D2 receptors presynaptically vs. postsynaptically, in various brain regions, or in their affinities, distribution, density, and tightness of coupling to a physiologic output.

Clinically, the term partial agonist may be misinterpreted because "partial" can imply weak or incomplete. DSSs, however, are not less effective than other types of antipsychotics. The prototypical member of this class aripiprazole has been found to reduce psychosis as effectively as other antipsychotics without causing motor side effects in schizophrenia(Kane et al. 2002; Ozdemir et al. 2002). Moreover, aripiprazole has been shown to have no adverse effect on prolactin levels, QTc prolongation, serum lipids and sugar and has a low potential for weight gain. All these beneficial characteristics has earned it a label of "Third generation antipsychotic".

The present study was to evaluate whether aripiprazole behaves similarly in the Indian population as in the western population and whether the recommended dose of 10-15mg/ day is safe and effective.

### METHOD:

A total of 7 centres in India participated in this open, randomized (to 10 or 15 mg aripiprazole), switch over multicentric study.

### Patients:

Eligible participants were male or female outpatients, 18 to 65 years of age, with diagnosis of schizophrenia as defined in ICD-10.

Categories of patients included:

1) Newly diagnosed
2) Patients who had achieved partial or no response to treatment with typical and atypical anti psychotics
3) Subjects who did not tolerate the prescribed antipsychotics.

Patients with history of more than one serious suicide attempt, organic brain disease and any other psychiatric disease, any chronic unstable medical illness and unwillingness to provide informed consent were excluded from the study. Patients taking epileptic medication and ECT, pregnant women, patients with history of repeated non-compliance with treatment and patients with clinically significant laboratory abnormality were also excluded from the study.

The study protocol was approved by the institutional review boards for all participating study centers and by regulatory authority of India. The patients gave a signed informed consent before any study-related procedure including screening could be undertaken.

### Study Design

The study was a multicentric trial, spread over diverse geographical locations in India. Patients underwent a comprehensive psychiatric and physical examination and appropriate hematology, biochemistry, urine pregnancy test (females) and ECG examination. Patients meeting eligibility criteria at screening were enrolled in the study. Category I patients were started on drug therapy immediately at a dose of 10 or 15 mg and continued at the same dosage for six weeks. Category II & III patients were switched to aripiprazole (10 or 15 mg/day) over a period of one week(switch strategy outlined below). The drug was continued on the randomized dose for a further 5 weeks.

**Switch Strategy**

Switch (for 7 days):

Aripiprazole (10 or 15 mg once daily) + previous drug tapered over one week (at same dose for day 1 to 3; reduced to half the dose for day 4 to 7, followed by discontinuation)

After switch (for 35 days):

Once daily dose 10 or 15 mg of Aripiprazole

**Follow-up visits**

Follow-up visits were conducted after 1,2, 3, 4, 5 & 6 weeks of treatment, during which efficacy and safety evaluations were conducted. Efficacy assessments at each visit included the PANSS scale, CGI-I and CGI-S scale. Adverse events were enquired and recorded at every visit.

Primary Efficacy Measures

1) PANSS Scale
2) CGI –S score and CGI –I score.
3) Response rate: > 30% decrease in PANSS score

**Safety and Tolerability Measures**

All adverse events volunteered, observed and enquired during the study or within 6 days of the last day of treatment were recorded together with their date of onset, duration, concurrent therapy, the investigator's assessment of severity, and the possible causative relationship to study drug, and whether a change in dose or withdrawal of treatment was required. A 12 lead ECG was done at baseline and at the end of the study. Clinical laboratory tests, including routine hematology, serum chemistry and liver function tests were carried out at baseline and at end of study.

**Statistical Analysis:**

Intention to Treat (ITT) statistical analysis has been performed on the data. Last Observation Carried Forward (LOCF) principle has been used for those patients who have taken the study drug for at least two weeks. Demographic data is presented in the form of Mean ± S.D. Significance between treatment groups for continuous type data has been subjected to Student's t-test & for Nominal type data has been subjected to Chi-Square test or Fisher Exact test. Since the data for PANSS is measured on ordinal scale the data for this parameter has been subjected to Non-Parametric test. The sum of ranks for all questions in PANSS score at respective visits was subjected to Wilcoxon sign rank test for finding the significance within the groups. Subjecting the data to ANCOVA with baseline value as its covariate tested significant difference between the treatment groups. Change in severity of illness (CGI-S) and global improvement (CGI-I) for both treatment groups from baseline to last visit was subjected to chi-square test for finding significance. Laboratory investigations data was first checked for its normality. If found normal, then the data was subjected to parametric test (paired T-test). Otherwise, the data was subjected to Non-Parametric (Wilcoxon Sing Rank) test for checking significance from baseline to last visit.

Significance between the treatment groups for interval type data has been subjected to student's t test. Nominal type data has been subjected to chi-square test. Fisher's exact test was used for categorical data where cell numbers were small. All the statistical tests used at 5% significance level.

**RESULTS**

**Demography**

A total of 136 patients were randomized (balance randomization) to receive either 10 or 15 mg/day of Aripiprazole in the study. Out of these,10 patients dropped out from the study before 2 weeks of the Aripiprazole Treatment. The dropouts have not been included in the analysis. Reasons for the drop outs are mentioned in the Table-3.

**DROP OUTS & WITHDRAWALS**

| Reasons | Total | Aripiprazole 10 mg | Aripiprazole 15 mg |
|---|---|---|---|
| AE's | 2 | 1 | 1 |
| Increased psychotic symptoms | 2 | 0 | 2 |
| Discontinuation on Pts. Request | 3 | 1 | 2 |
| Poor compliance | 1 | 0 | 1 |
| Lost to follow-up | 2 | 1 | 1 |
| Total | 10 | 3 | 7 |

Note: patient mentioned more than one reasons

Twenty patients were lost to follow-up after completing more than 2-weeks of aripiprazole treatment and have been included in the analysis using the statistical technique of last observation carried forward (LOCF). Out of the 126 patients who have been analysed, 65 patients were taking 10 mg aripiprazole once daily and 61 were taking 15 mg aripiprazole once daily.

The demographic data of patients who were analysed is presented in Table -1.

Table 1.

**DEMOGRAPHIC PROFILE OF PATIENTS**

| | Total (n=126) | 10 mg (n=65) | 15 mg (n=61) |
|---|---|---|---|
| Age (years) | 34 ± 10 | 35±11 | 33±9 |
| Males (n) | 80 | 43 | 37 |
| Females (n) | 46 | 22 | 24 |
| Duration of illness (median months) | 49 months | 42 months | 36 months |
| History of ≤1 suicide attempt (n) | 5 | 4 | 1 |
| Category of Patients | | | |
| Newly diagnosed (n) | 29 | 18 | 11 |
| Intolerant to previous therapy (n) | 16 | 6 | 10 |
| Partial or non-responders (n) | 81 | 41 | 40 |

The average age of the patients was 34±10 years. There were more number of males than females in the study. The median duration of illness was around 4 years. Three categories of patients were enrolled as per the inclusion criteria. There were 29 newly diagnosed patients, 16 patients were intolerant to previous therapy and 81 patients were partial-responder or non-responders to previous therapy. The patients were equally distributed in the 10 mg & 15 mg dose groups as regards age, sex, duration of illness and category of patients.

Therapies from which the patients were switched to aripiprazole included both typical and atypical antipsychotic drugs

Table: 2.

**Therapies from which the patients were switched to aripiprazole**

| Drug Name | No. of Patients |
|---|---|
| Chlorpromazine | 7 |
| Clozapine | 20 |
| Flupenthixol | 6 |
| Haloperidol | 28 |
| Olanzapine | 50 |
| Quetiapine | 5 |
| Risperidone | 68 |
| Trifluperazine | 38 |
| Ziprasidone | 14 |

Note: Many patients were on more than one anti-psychotic drug.

Many patients were on more than one antipsychotic drug. Most commonly administered previous therapies included olanzapine , risperidone, haloperidol and trifluperazine.

**Efficacy Evaluation:**

Change in total PANSS score (from baseline) was measured as the Efficacy end point for the Patients. Aripiprazole treatment, in the dose of 10 and 15 mg for a period of 6-weeks, consistently decreased total PANSS score over the study duration. The decrease in PANSS score (compared to baseline) reached statistical significance (P<0.001) from week-2 (Figure-1).

Figure 1.

**Change in Total PANSS - Score Over 6-Weeks of Aripiprazole (10 mg /15 mg) Treatment**

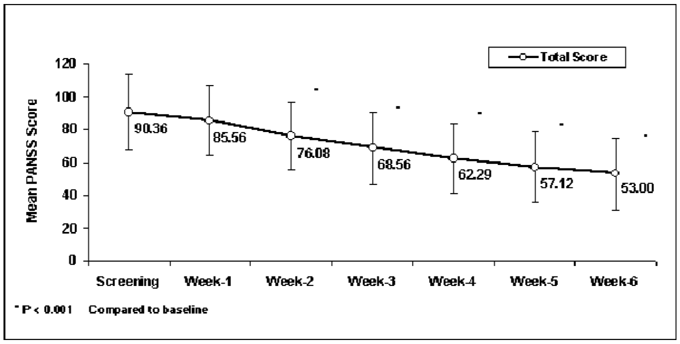

Aripiprazole produced a similar effect on the Positive and Negative sub-scale scores of PANSS. Both the sub-scale scores showing significant reduction over baseline, starting from week-2(Figure-2).

Figure 2.

**Change in Sub-Scales Scores of PANSS by Aripiprazole (10 mg/ 15 mg) Treatment Over 6-Weeks**

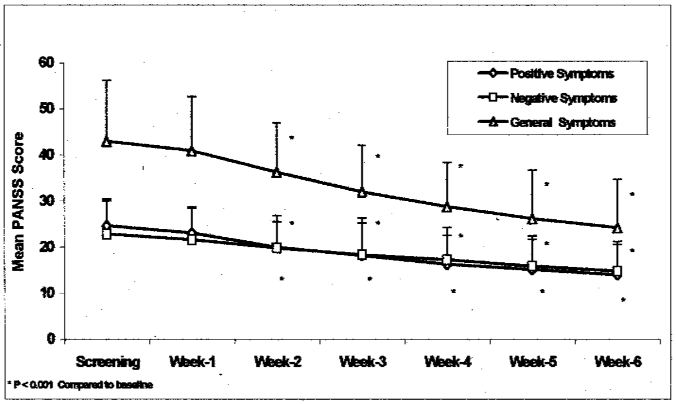

On comparison of two dose groups as regards the reduction in the PANSS scores it was seen that there was no significant difference in the two treatment groups (10mg and 15 mg)( Figure-3).

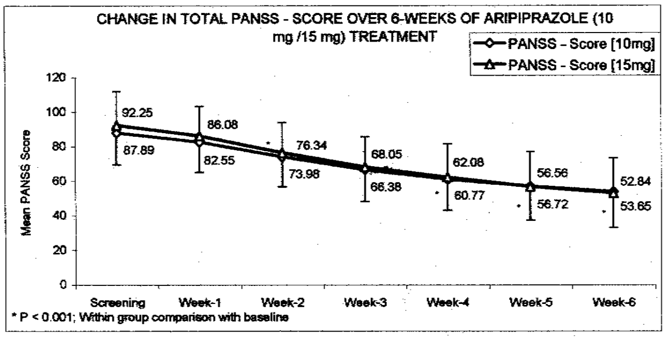

CGI-S scores improved over 6-week treatment with aripiprazole showing thereby a decrease in the disease severity (Figure-4).

Figure 4:

**Change In CGI-S Scores Over 6-Weeks Of Treatment With Aripiprazole(10 mg &15 mg)**

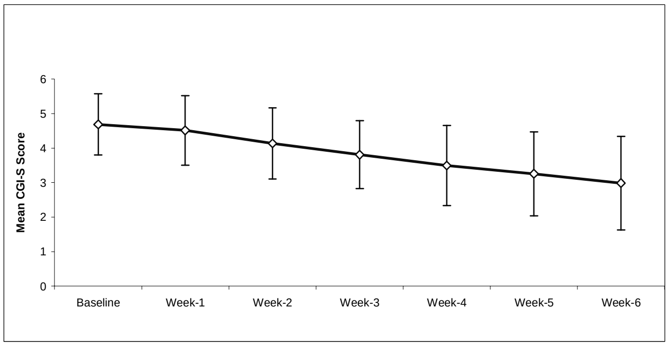

Both the dose groups (10 mg and 15 mg) showed equal improvement in the CGI-S scores (Figure-5).

Figure 5:

**Comparison of change in CGI-S scores by Aripiprazole in the 10 mg dose group with 15 mg dose group**

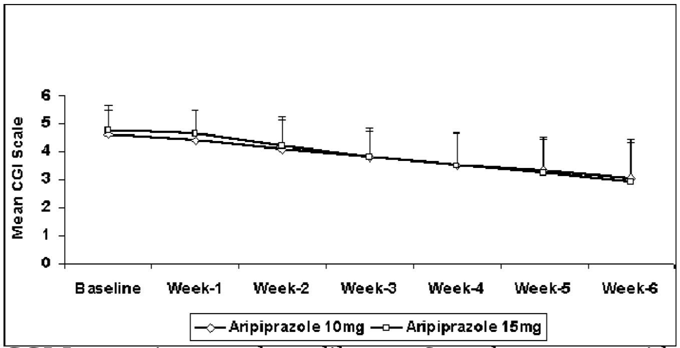

CGI-I scores improved steadily over 6-week treatment with aripiprazole, a reflection of the global improvement in the symptomatology of the patients (Figure-6).

Both the dose groups (10 mg and 15 mg) showed equal improvement in the CGI-I scores (Figure-7).

Figure 6.

**Change In Mean CGI-I Scores Over 6-Weeks Of Treatment With Aripiprazole(10 mg &15 mg)**

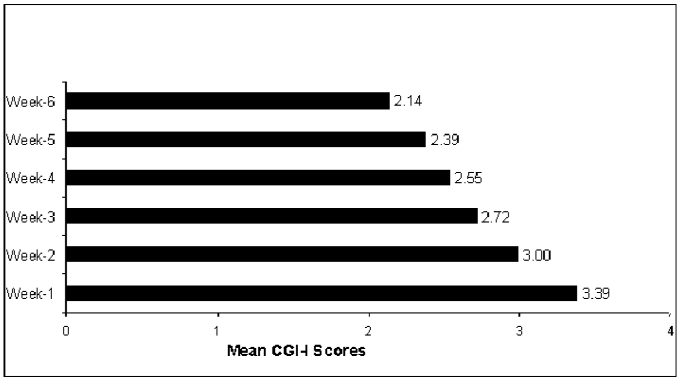

Figure 7.

**Comparison of change in CGI-I scores by Aripiprazole in the 10 mg dose group with 15 mg dose group**

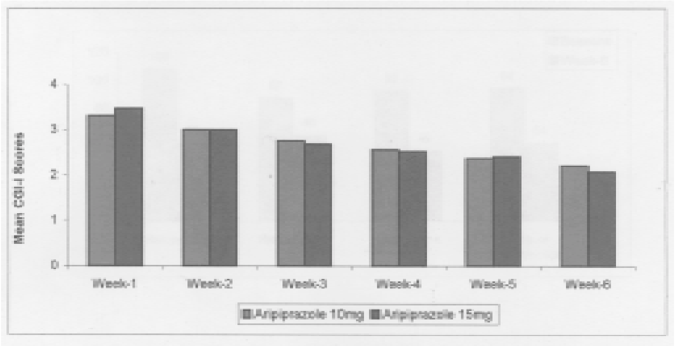

Response rate (decrease in PANSS scores by ≥30%) to aripiprazole after 6-weeks of therapy was more than 70% in both the dose groups (10 mg and 15 mg) (Figure-8).

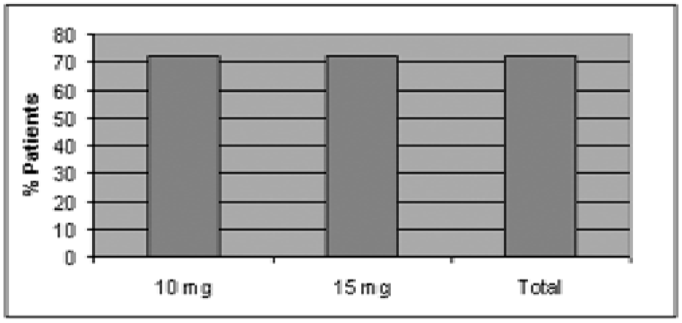

Switching schizophrenic patient to aripiprazole (10 mg and 15 mg) from both conventional (haloperidol, trifluperazine) and atypical anti-psychotics (risperidone, olanzapine, quetiapine) produced improvement in the symptomatology over previous therapies. This is evident by the improvement in PANSS score and CGI-I score over baseline (Figure-9& 10).

Safety Evaluation:

No unexpected adverse event was reported. About 70% of patients reported at least one adverse event during the

Figure 9:

**Change in PANSS score on Switching to Aripiprazole**

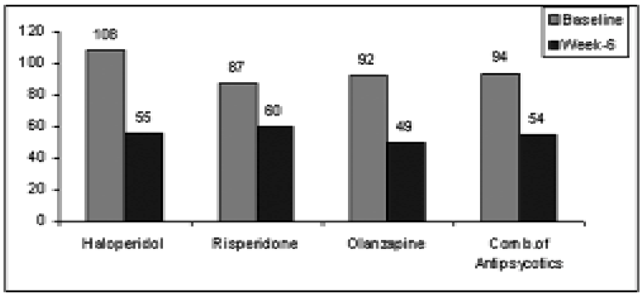

Figure 10:

**Change in CGI-I Score on Switching to Aripiprazole**

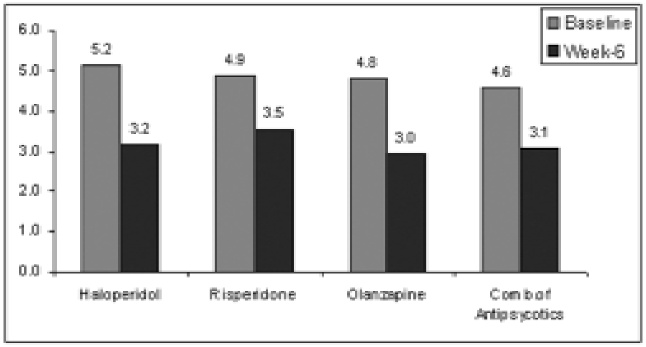

6-weeks of treatment with aripiprazole (Table-4). 15-mg dose group showed a slightly higher proportion of adverse events (72%) as compared to 10-mg dose group (66%).

The most commonly reported adverse events were fatigue

Table 4.

**ADVERSE EVENTS (AES)**

| Major AE's observed | Total (n=126) | 10 mg Aripiprazole (n=65) | 15 mg Aripiprazole (n=61) |
|---|---|---|---|
| Patient having AE's n (%) | 87(69%) | 43(66%) | 44(72%) |
| Dropout due to AE's n (%) | 2 (2%) | 1(1%) | 1(1%) |
| Fatigue | 27(32%) | 13(20%) | 14 (23%) |
| Insomnia | 24(27%) | 13(20%) | 21 (34%) |
| Headache | 21(24%) | 16(24%) | 13(21%) |
| Nausea / Vomiting | 17(19%) | 14(22%) | 8(13%) |
| Tremor | 18(20%) | 7(11%) | 9(14%) |
| Rigidity | 4(5%) | 3(5%) | 1(1%) |
| Akathisia | 4(5%) | 2(3%) | 2(3%) |
| Dyskinesia | 0(0%) | 0(0%) | 1(1%) |
| Decrease in libido | 1(1%) | 0(0%) | 1(1%) |
| Constipation | 10(12%) | 7(11%) | 3(5%) |
| Blurred vision | 1(1%) | 0(0%) | 1(1%) |
| Hepatitis | 1(1%) | 1(1%) | 0(0%) |
| Other AE's | 33(37%) | 16(25%) | 17(28%) |

(32%), insomnia (27%), headache (24%), nausea/vomiting (19%), tremor (20%), rigidity (5%), akathisia (5%), constipation (12%). There was no significant difference in the two dose groups.

No clinically relevant change was observed after 6-weeks

Table 5 : **Laboratory Investigations at baseline and after 6 weeks of treatment**

| **Lab Parameters** | **At Screening (n=126)** | | **At Week-6 (n=106)** | | **Significance e** |
|---|---|---|---|---|---|
| | **Mean** | **S.D.** | **Mean** | **S.D.** | **(P Value)** |
| QTc Interval | 400.46 | 43.39 | 396.78 | 49.69 | 0.456 |
| Hb (gm%) | 12.89 | 1.53 | 12.73 | 1.49 | 0.453 |
| RBC-Total count (cell/mm3) | 4.67 | 0.66 | 4.69 | 0.59 | 0.866 |
| WBC-Total Amount (cell/mm3) | 7214.18 | 1446.38 | 7463.83 | 1487.05 | 0.211 |
| WBC-DC (N/P) | 64.73 | 7.11 | 65.81 | 7.45 | 0.862 |
| WBC-DC (L) | 29.72 | 6.70 | 28.43 | 6.49 | 0.534 |
| WBC-DC (M) | 3.48 | 2.34 | 4.10 | 2.24 | 0.883 |
| WBC-DC (E) | 2.84 | 2.55 | 2.84 | 2.72 | 0.350 |
| WBC-DC (B) | 3.94 | 2.14 | 2.00 | 0.00 | — |
| SGOT (U) | 29.34 | 10.67 | 26.13 | 9.90 | 0.016 |
| SGPT (U) | 29.89 | 13.58 | 29.28 | 14.11 | 0.970 |
| Serum alkaline phosphatase (U) | 117.06 | 78.23 | 107.56 | 74.10 | 0.928 |
| Total bilirubin (mg%) | 0.58 | 0.21 | 0.52 | 0.20 | 0.022 |
| Serum Creatinine (mg%) | 0.78 | 0.26 | 0.70 | 0.30 | 0.023 |
| Blood Urea (mg%) | 21.99 | 6.21 | 21.07 | 6.33 | 0.388 |
| Blood Sugar (Fasting) | 93.86 | 30.21 | 86.70 | 23.45 | 0.106 |
| Prolactin | 18.94 | 17.09 | 9.65 | 6.66 | 0.000 |
| Serum Cholesterol | 187.84 | 40.64 | 179.43 | 38.04 | 0.094 |
| Serum Triglycerides | 139.99 | 77.54 | 136.87 | 75.44 | 0.603 |
| Serum HDL Cholestrol | 44.29 | 10.52 | 49.88 | 32.30 | 0.268 |
| Serum LDL Cholestrol | 99.92 | 39.09 | 100.62 | 32.84 | 0.696 |

of aripiprazole treatment in the laboratory parameters like QTc interval, hematology, Serum biochemistry and plasma lipid profile(Table-5). However, there was a significant reduction (from baseline) in prolactin levels after aripiprazole therapy(P<0.0001).

There was a non-significant decrease in the mean body weight after 6-weeks of aripiprazole therapy (10 mg and 15 mg)(Figure-11).

Figure 11:

**Change in Weight after 6-weeks of treatment with Aripiprazole (10 mg & 15 mg)**

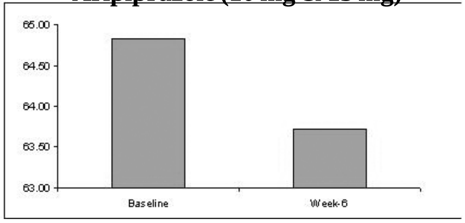

Around 50% of patients had no change in the body weight, 40% had a weight loss over baseline, 10% had a weight gain and 2% had significant weight gain(more than 7% increase in body weight) (Figure-12 &13).

Figure 12:

**Changes in the Body Weight in Patients After 6-weeks of Aripiprazole Therapy**

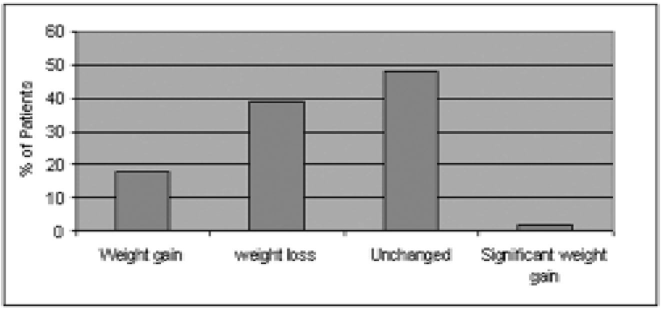

Figure 13: **Change in Weight on Switching to Aripiprazole**

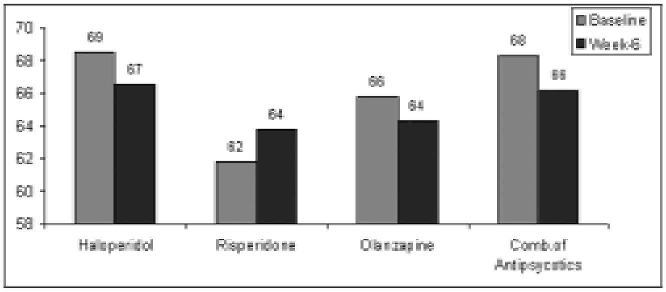

Most of the adverse events reported with aripiprazole (10 mg and 15 mg) were mild to moderate in severity, were not serious, did not require discontinuation of therapy, required no treatment, were transient in nature and were unlikely to be related to the study drug(Figure-14 & 15).

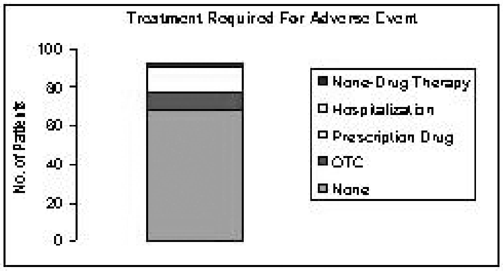

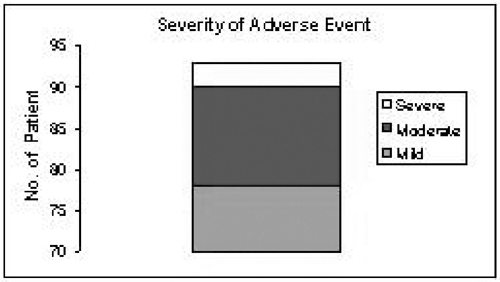

Patients showing side effects at the baseline with the previous therapies showed improvement with the aripiprazole treatment(10 mg and 15 mg)(Table-6).

| Side Effects | Baseline | Week-6 |
|---|---|---|
| Amennorhea | 2 | 1 |
| Drowsiness | 2 | 0 |
| Dry mouth | 1 | 0 |
| Tremor | 7 | 2 |
| Rigidity | 3 | 0 |
| Body Ache | 1 | 0 |
| Sedation | 1 | 0 |
| Total | 17 | 3 |

### DISCUSSION:

Classical neuroleptics, known as conventional antipsychotics, bind to $D_2$ receptors throughout the brain as powerful, long-acting antagonists. Unfortunately, the blocking is often nonspecific, which is typical of conventional antipsychotic actions. Thus, when D2 receptors in nigrostriatal pathway are also blocked, a penalty is paid in motor side effects, namely extrapyramidal reactions, pseudoparkinsonism, and ultimately, tardive dyskinesia.

Ever since the motor complications of conventional antipsychotics were recognized attempts have been made to preserve their motor side effects. Second generation antipsychotics, known as atypical antipsychotics, bind better to $D_2$ receptors in parts of the brain that control psychosis than in parts of the brain that causes motor side effects.

Such differential binding is a consequence of reduction of $D_2$ receptor antagonism where it is not desired, either by simultaneous blockade of 5-$HT_{2A}$ receptors, or by short-acting blockade at $D_2$ receptor, called "hit-and-run" antagonism. For these reasons, atypical antipsychotics have become the current standard of treatment of schizophrenia. However, there are still some aspects of the atypical antipsychotics which call for improvement, including — QT prolongation by ziprasidone; increase in serum prolactin by risperidone; deranged lipid profile by olanzapine and quetiapine; weight gain by olanzapine, quetiapine and risperidone; nonselective receptor binding (High muscarinic receptor affinity of olanzapine, high alfa-adrenergic receptor affinity of risperidone, olanzapine, quetiapine and ziprasidone),

Third-generation antipsychotics are dopamine system stabilizers (DSSs). They block $D_2$ receptors sufficiently where dopamine activity needs to be reduced (mesolimbic pathway), and thereby produce an antipsychotic action. However, DSSs do not simultaneously reduce dopamine activity in those brain regions where normal dopamine levels those brain where normal dopamine levels are needed (nigrostriatal pathway) and thus do not cause motor side effects. DSSs may even provide a modest boost in dopamine activity in areas of the brain where it needs to be increased (mesocortical pathway), and thus improve the negative and cognitive symptoms of schizophrenia.

Aripiprazole is a potentially novel antipsychotic with a mechanism of action that differs from the currently marketed typical and atypical antipsychotics. Biochemically, aripiprazole has been shown to be a partial agonist at members of the D2 family of dopamine receptors (Lawler et al. 1999; Burris et al 2002). In addition, Aripiprazole has been shown to exhibit partial agonists activity on the spontaneous release of prolactin from isolated rat anterior pituitary slices (Burris et al 2002). In vivo, Aripiprazole has been shown to exhibit antagonistic properties in animal models of dopaminergic hyperactivity (Ozdemir et al. 2002). Previously reported phase – II and III clinical data demonstrated Aripiprazole to be superior to placebo for improving PANSS total score (Ozdemir et al. 2002). All known effective antipsychotic act at $D_2$ receptors. A novel concept for an antipsychotic without motor side effects is to stabilize these receptors rather than block them harshly.

The present switch-over study showed that switching schizophrenic patient to aripiprazole from both conventional and atypical anti-psychotics is safe and well tolerated. Switching to aripiprazole over one week with tapering dose of previous drug was safe and well tolerated.

Aripiprazole offers advantages over presently available typical and atypical antipsychotic drugs. Six weeks after switching to aripiprazole, patients showed improvements in PANSS scores, CGI-I scores, Extra Pyramidal Symptoms, prolactin levels and weight over previous therapies.

Aripiprazole was effective against both positive and negative symptoms of schizophrenia.

The two dose groups 10 mg and 15 mg showed equal efficacy and safety.

**Safety summary:**

No serious or unexpected adverse event was reported during the study. Most common side effects were insomnia, fatigue, tremor and headache.

Most of the adverse events reported with aripiprazole (10 mg and 15 mg) were mild to moderate in severity, were not serious, did not require discontinuation of therapy, required no treatment, were transient in nature and were unlikely to be related to the study drug.

No significant adverse effect was observed on QTc prolongation, prolactin, serum lipids, renal function and hepatic function.

Aripiprazole has low potential for Extra Pyramidal Symptoms as evidenced by a very low rate of EPS in the study. It also has a low potential for weight gain.

The findings of the study are similar to the findings reported in the literature both in terms of efficacy and safety (McGavin et al. 2002; Drugs 2002). Switch over studies are becoming an important tool for evaluating new drugs because of their closeness to the clinical situations. They are also more acceptable to the patients because of the lack of a placebo arm.

### REFERENCES

Aripiprazole(2002) Drugs, 3(1): 25-27.

Burris K.D., Molski T.F. et al.(2002) Aripiprazole, a Novel Antipsychotic, Is a High-Affinity Partial Agonist at Human Dopamine D2 Receptors, JPET, 302:381-89.

Kane M.J. , Carson W.H., et al. (2002) Efficacy And Safety Of Aripiprazole And Haloperidol Versus Placebo In Patients With Schizophrenia And Schzioaffective Disorder, J Clin Psychiatry, 63:763-771.

Lawler P.C., Prioleau C, et al.(1999) Interactions of the Novel Antipsychotic Aripiprazole (OPC-14597) with Dopamine and Serotonin Receptor Subtypes, Neuropsychopharmacology, 20: 612-627.

McGavin K.J. & Goa L.K. (2002) Aripiprazole, Drugs, 16(11):779-786.

Ozdemir V, Fourie J & Ozdener F (2002) Aripiprazole, Current Opinion in Investigational Drugs, 3(1):113-120.

Stahl S.M. (2001) Dopamine System Stabilizers, Aripiprazole, And The Next Generation Of Antipsychotics, Part 2 Illustrating Their Mechanism Of Action, J Clin Psychiatry, 62:923-924.

Stahl S.M. (2001) Dopamine System Stabilizers, Aripiprazole, And The Next Generation Of Antipsychotics, Part 1 "Goldilocks" Actions At Dopamine Receptors , J Clin Psychiatry, 62:811-812.
